# Supplementary material for: An analysis of the impact of China’s macroeconomic performance on its trade partners: Evidence based on the GVAR model
Source: PLoS One. 2023 Jan 3;18(1):e0275859. doi: 10.1371/journal.pone.0275859 (PMC9810200; doi:10.1371/journal.pone.0275859)
Supplement: Attachment — Submitted filename: Response to reviewers.docx [file pone.0275859.s002.docx]

**Response to Editor:**

1. Journal Requirements: We are thankful for considering our paper in your prestigious journal. We have revised our manuscript to meets PLOS ONE's style requirements.
2. Captions for Supporting Information files have been included at the end of manuscript.

**Response to Reviewers' comments:**

1. The paper has been revised and emphasis been made on the value added in section 3.8 and section 4. Besides this, the novelty of the paper and the findings of our study have been compared and contrasted with the existing literature in section 3.8.
